# Supplementary material for: Immunoglobulin G Subclass-Specific Glycosylation Changes in Rheumatoid Arthritis
Source: Int J Mol Sci. 2025 Oct 2;26(19):9626. doi: 10.3390/ijms26199626 (PMC12525468; doi:10.3390/ijms26199626)
Supplement: Supplementary file 1 [file ijms-26-09626-s001.zip › ijms-3862394-supplementary.pdf]

Supplementary Table S1. The list of investigated N-glycans

IgG Isoform	Glycan	Glycosylation parameters			
		Galactosylation	Sialylation	Fucosylation	Bisecting
	N4H3	0	0	0	0
	N4H4	1	0	0	0
	N4H5	2	0	0	0
	N4H3F1	0	0	1	0
	N4H4F1	1	0	1	0
	N4H5F1	2	0	1	0
	N5H5F1	2	0	1	1
	N4H4S1	1	1	0	0
	N4H5S1	2	1	0	0
	N4H4S1F1	1	1	1	0
	N4H5S1F1	2	1	1	0
	N5H5S1F1	2	1	1	1
	N5H3F1	0	0	1	1
	N3H4F1	1	0	1	0
	N3H4S1F1	1	1	1	0
	N5H3	0	0	0	1
	N5H4	1	0	0	1
	N5H4F1	1	0	1	1
	N5H4S1F1	1	1	1	1
	N4H3	0	0	0	0
	N4H4	1	0	0	0
	N4H5	2	0	0	0
	N4H3F1	0	0	1	0
	N4H4F1	1	0	1	0
	N4H5F1	2	0	1	0
	N5H5F1	2	0	1	1
	N4H4S1	1	1	0	0
	N4H5S1	2	1	0	0
IgG2	N4H4S1F1	1	1	1	0
	N4H5S1F1	2	1	1	0
	N5H5S1F1	2	1	1	1
	N5H3F1	0	0	1	1
	N3H4F1	1	0	1	0
	N3H4S1F1	1	1	1	0
	N5H3	0	0	0	1
	N5H4	1	0	0	1
	N5H4F1	1	0	1	1
	N5H4S1F1	1	1	1	1
IgG3/4	N4H3F1	0	0	1	0

N4H4F1	1	0	1	0
N4H5F1	2	0	1	0
N5H5F1	2	0	1	1
N4H4S1F1	1	1	1	0
N4H5S1F1	2	1	1	0
N5H3F1	0	0	1	1
N5H4F1	1	0	1	1
